# Transcriptional response of the model planctomycete *Rhodopirellula baltica *SH1^T ^to changing environmental conditions

**DOI:** 10.1186/1471-2164-10-410

**Published:** 2009-09-02

**Authors:** Patricia Wecker, Christine Klockow, Andreas Ellrott, Christian Quast, Philipp Langhammer, Jens Harder, Frank Oliver Glöckner

**Affiliations:** 1 Microbial Genomics Group, Max Planck Institute for Marine Microbiology, Microbial Genomics Group, Celsiusstr. 1, 28359 Bremen, Germany; 2 Department of Microbiology, Max Planck Institute for Marine Microbiology, Microbiology, Celsiusstr. 1, 28359 Bremen, Germany; 3 Jacobs University Bremen gGmbH, Campusring 1, 28759 Bremen, Germany

## Abstract

**Background:**

The marine model organism *Rhodopirellula baltica *SH1^T ^was the first *Planctomycete *to have its genome completely sequenced. The genome analysis predicted a complex lifestyle and a variety of genetic opportunities to adapt to the marine environment. Its adaptation to environmental stressors was studied by transcriptional profiling using a whole genome microarray.

**Results:**

Stress responses to salinity and temperature shifts were monitored in time series experiments. Chemostat cultures grown in mineral medium at 28°C were compared to cultures that were shifted to either elevated (37°C) or reduced (6°C) temperatures as well as high salinity (59.5‰) and observed over 300 min. Heat shock showed the induction of several known chaperone genes. Cold shock altered the expression of genes in lipid metabolism and stress proteins. High salinity resulted in the modulation of genes coding for compatible solutes, ion transporters and morphology. In summary, over 3000 of the 7325 genes were affected by temperature and/or salinity changes.

**Conclusion:**

Transcriptional profiling confirmed that *R. baltica *is highly responsive to its environment. The distinct responses identified here have provided new insights into the complex adaptation machinery of this environmentally relevant marine bacterium. Our transcriptome study and previous proteome data suggest a set of genes of unknown functions that are most probably involved in the global stress response. This work lays the foundation for further bioinformatic and genetic studies which will lead to a comprehensive understanding of the biology of a marine *Planctomycete*.

## Background

Marine ecosystems, covering approximately 71% of the Earth's surface, host the majority of biomass and contribute significantly to global cycles of matter and energy. Microorganisms are known to be the 'gatekeepers' of these processes, and insight into their lifestyle and fitness enhances our ability to monitor, model and predict the course and effect of global changes. Nevertheless, specific knowledge about their functions is still sparse. The 'genomic revolution' [1] has opened the door to investigations targeting their genetic potential and activity on the molecular level.

A particularly interesting representative of the marine picoplankton community is *Rhodopirellula baltica *SH1^T^, a free-living bacterium which was isolated from the water column of the Kiel Fjord (Baltic Sea) [2]. *R. baltica *belongs to the phylum *Planctomycetes*, a broadly distributed group of bacteria, whose members can be found in terrestrial, marine and freshwater habitats [3-7], but also in extreme environments like hot springs [8], marine sponges [9] and the hepatopancreas of crustaceans [10].

In terms of cell biology all *Planctomycetes *share several morphologically unique properties, such as a peptidoglycan-lacking proteinaceous cell wall [11,12], intracellular compartmentalization [13] and a mode of reproduction via budding. The latter results in a cell cycle that is characterized by motile and sessile morphotypes similar to *Caulobacter crescentus *[14-17]. A specific holdfast substance produced by sessile cells allows *R. baltica *to attach to macroscopic detrital aggregates (marine snow) [3,7].

At present, four planctomycete genomes are currently available [18]. Of these, the genome of *R. baltica *is the only one completely closed [16]. The genome was found to be 7,145,576 bases in size and codes for 7325 open reading frames (ORFs) plus 72 RNA genes. Originally, only 45% of the ORFs were assigned particular functions [16]. Thus, over 55% of all proteins in the genome remain functionally uncharacterized. These were referred to as 'hypothetical proteins' with or without the affix 'conserved' contingent on wider phylogenetic distribution [19]. A subset of these conserved hypothetical proteins is specific for *Planctomycetes *[18]. It seems likely that some of these genes code for the unique planctomycetal cellular characteristics and metabolic traits.

The availability of the genome information triggered several key post-genomic studies including studies of the proteome [20-24], enzyme activity [25] and protein crystallization [26].

In summary, these studies confirmed the hypothesis of Glöckner *et al*. that *R. baltica *is a polysaccharide degrader [16]. It appears *R. baltica *is gaining carbon and energy from the decomposition of complex heteropolysaccharides originally produced by algae in the photic zone while slowly sedimenting with the marine snow.

Marine microorganisms like *R. baltica *are exposed to rapidly changing environmental conditions such as varying temperature, salinity, irradiance and oxygen concentration. Typically, sudden changes of these environmental conditions induce a stress response in the exposed planktonic community characterized by a distinct change in their gene expression pattern. This stress response enables the organisms to protect vital processes and to adapt to the new condition. Such responses have been described for a set of organisms from different environments including *Shewanella oneidensis *[27,28], *Pseudomonas aeruginosa *[29], *Desulfovibrio vulgaris *Hildenborough [30], *Xylella fastidiosa *[31], *Synechocystis *sp. [32] and Yeast [33].

To gain insights into the stress responses of *R. baltica *with respect to salinity and temperature the first whole genome array for *R. baltica - *also the first *Planctomycete *microarray - was established and applied. The reported data will serve as a resource to expand our understanding of the physiological and transcriptional response of *R. baltica *to the wide range of changing environmental conditions a free-living marine bacterium is exposed to.

## Results and Discussion

### Overview

**Figure 1:**
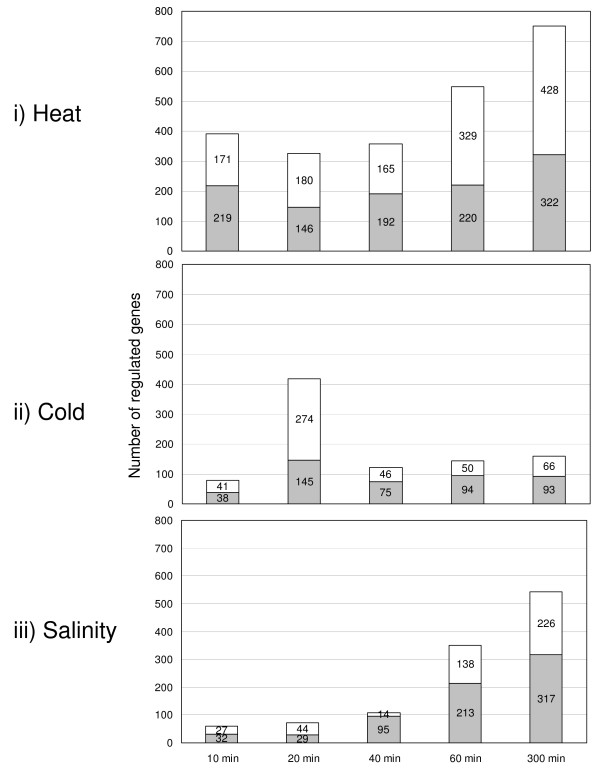
**Number of regulated genes per stress experiment**. Columns show the total number of up- (gray) and down- (white) regulated genes at each sampled time point compared to reference samples. i) heat shock, ii) cold shock and iii) high salinity

54 distinct, total RNA samples were analyzed by whole-genome microarray hybridization. Differential expression of 2372, 922 and 1127 genes was noted during heat shock, cold shock and salt stress, respectively, at one or more of the five time points when compared to reference samples (FIGURE 1i; ii &1iii). With only 45% of the genes in *R. baltica's genome *functionally annotated, it is not surprising that most of the differentially expressed genes were hypothetical or conserved hypothetical proteins. The complete list of the differentially expressed genes for each shift experiment and time point is available in the ADDITIONAL FILE 1.

**Figure 2:**
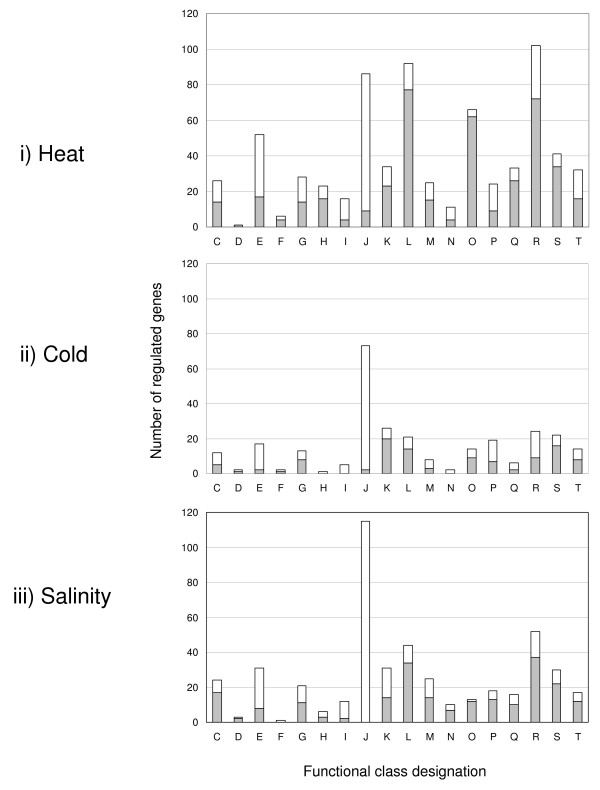
**Number of regulated genes with an assigned COG-category**. Columns show the number of up- (gray) and down- (white) regulated genes per assigned COG-category according to the NCBI database (cut off e-value e-4). i) heat shock, ii) cold shock and iii) high salinity; Columns: [C] Energy production and conversion, [D] Cell division and chromosome partitioning, [E] Amino acid transport and metabolism, [F] Nucleotide transport and metabolism, [G] Carbohydrate transport and metabolism, [H] Coenzyme metabolism, [I] Lipid metabolism, [J] Translation, ribosomal structure and biogenesis, [K] Transcription, [L] DNA replication, recombination and repair, [M] Cell envelope biogenesis, outer membrane, [N] Cell motility and secretion, [O] Posttranslational modification, protein turnover, chaperones, [P] Inorganic ion transport and metabolism, [Q] Secondary metabolites biosynthesis, transport and catabolism, [R] General function prediction only, [S] Function unknown, [T] Signal transduction mechanisms

**Figure 3:**
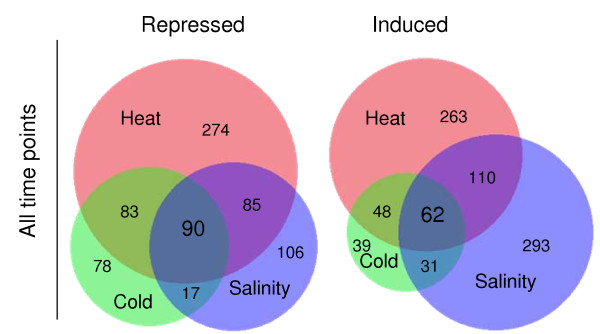
**Venn diagrams of specific and common stress response**. The diagram shows the distribution of stimulus-specific and common stress responses. All genes of all time points are represented in one diagram divided into repressed and induced genes.

Only 32% of the regulated genes in the heat and cold shock experiments could be assigned with a COG function (FIGURE 2i &2ii) while 37% were assignable in the salt stress experiment (FIGURE 2iii). This is in line with the 36% (2661 genes) of COG functional class designations in *R. baltica*. A striking feature of the expression profiles displayed is the stereotypical response of a large fraction of the genome to all three stress conditions. In summary, 152 genes are up- or down-regulated at any time point for all stressors. Of these 152 genes, 62 are induced and 90 are repressed (TABLE 1 and TABLE 2). 49% of the induced and 61% of the repressed genes were annotated as hypothetical proteins. The Venn diagrams shown in FIGURE 3 provide an overview of the specific and common genes of the three stress-specific responses. To identify co-regulated patterns of gene expression, we classified all differentially expressed genes of all three stress expressions into 30 k-means clusters based on their expression log ratio. To determine the necessary number of clusters a figure of merit was generated. 30 clusters were considered as adequate. The cluster data are available in the ADDITIONAL FILE 2. Clusters 1, 3 and 4 show a similar response to the specific environmental changes, called environmental stress response (ESR) over all experiments. Clusters 2, 4, 5, 7, 15 and 22 describe genes reacting to a specific environmental factor.

**Table 1:** Shared stress response to heat, cold and high salinity: Results for induced genes are shown

**ID**	**AA**	**Product**	**IEP**	**Strand**	**Potentially involved in/Comments**
RB170	96	Transposase IS3/IS911	10.1	+	
RB370	553	nitrate transporter substrate-binding protein	4.6	+	
RB521	63	hypothetical protein	10.7	-	[24,66]
RB723	60	hypothetical protein	7.3	+	
RB934	375	Putative transposase	9.9	-	
RB1394	78	hypothetical protein	10.1	-	regulatory mechanism
RB1395	319	secreted protein similar to DNA-binding protein	5.5	+	regulatory mechanism
RB1789	243	conserved hypothetical protein	9.4	+	regulatory mechanism
RB1872	38	hypothetical protein	12.3	+	
RB2186	433	ISXo8 transposase	9.4	-	
RB2268	282	peptide methionine sulfoxide reductase	9.9	-	[22]
RB3596	144	nitrogen fixation protein (NifU protein)	4.3	+	
RB4299	96	Transposase IS3/IS911	9.9	+	
RB4347	156	conserved hypothetical protein	4.7	+	[24,66]
RB4397	55	protein containing DUF1560	10.5	+	
RB4429	89	conserved hypothetical protein	4.9	+	stress response
RB4433	162	Ferritin and Dps	4.3	+	
RB4438	160	Pyridoxamine 5'-phosphate oxidase-	4.4	+	
RB4510	49	hypothetical protein	9	-	
RB5238	73	hypothetical protein	10.6	+	
RB5551	663	hypothetical protein	5.7	+	DVL-domain
RB5888	96	Transposase IS3/IS911	10.1	-	
RB5938	370	hypothetical protein-	5.6	-	[66]
RB6928	160	hypothetical protein	4.1	+	
RB7389	375	Putative transposase	9.9	+	
RB8409	97	hypothetical protein	8.9	+	
RB8527	330	protein containing DUF1559	6.6	+	stress response
RB8987	48	hypothetical protein	9.1	-	
RB9230	107	hypothetical protein	9.9	+	next to transposase
RB9907	433	ISXo8 transposase	9.4	+	
RB9955	452	secreted protein containing DUF1552	5.6	-	regulatory mechanism
RB9999	281	conserved hypothetical protein-	4.6	-	regulatory mechanism
RB10049	217	RNA polymerase ECF-type sigma factor	10.1	+	
RB10378	144	Thioredoxin	4.6	-	
RB10727	276	manganese-containing catalase	5	+	[24,66]
RB10728	132	secreted protein	9.9	+	stress response
RB10896	161	secreted protein	10	-	stress response
RB10954	143	hypothetical protein	10.4	-	
RB10956	117	hypothetical protein	4.8	+	[24]
RB10957	99	conserved hypothetical protein	5.6	+	regulatory mechanism
RB10958	158	hypothetical protein	5.4	+	
RB11176	153	protein containing DUF442	4.8	-	[24] stress response
RB11260	121	dnaK suppressor protein,	5.2	-	
RB11475	57	conserved hypothetical protein	4.7	+	next acyltranscferase, short protein
RB11504	72	conserved hypothetical protein	10.7	-	short protein,
RB11505	199	conserved hypothetical protein, secreted	7.5	-	
RB11515	74	conserved hypothetical protein	11.7	+	
RB11566	195	hypothetical protein	10.8	+	
RB11749	96	Transposase IS3/IS911	10.1	+	
RB11750	292	integrase	10	+	
RB11802	96	Transposase IS3/IS911	10.1	+	
RB11855	101	conserved hypothetical protein	12.5	-	
RB11918	134	protein containing DUF971	6.1	-	
RB11977	196	conserved hypothetical protein	9.5	+	
RB12066	135	hypothetical protein	10	+	
RB12239	433	ISXo8 transposase	9.4	+	
RB12247	74	conserved hypothetical protein	6.3	-	
RB12936	580	conserved hypothetical protein	5.3	-	DUF 444
RB12940	96	Transposase IS3/IS911	10.1	+	
RB13222	208	SOUL heme-binding protein	8.7	-	
RB13241	167	RNA polymerase ECF-type sigma factor	8.8	-	

**Table 2:** Shared stress response to heat, cold and high salinity: Results for repressed genes shown

**ID**	**AA**	**Product**	**IEP**	**Strand**	**Potentially involved in/Comments**
RB61	58	hypothetical protein	9.6	+	
RB314	309	malonyl CoA-acyl carrier protein transacylase	4.4	+	
RB318	81	Acyl carrier protein	3.7	+	
RB319	95	hypothetical protein	10.2	+	fatty acid process
RB767	311	conserved hypothetical protein, secreted	5.7	+	
RB825	117	hypothetical protein	7.8	-	
RB951	234	protein containing DUF1596	12.3	-	
RB1129	895	conserved hypothetical protein	5.8	-	(S) bombinin, defense response
RB1233	206	30S ribosomal protein S4	11.2	-	
RB2105	470	membrane protein	9.6	-	
RB2306	41	hypothetical protein	9	+	
RB2479	273	conserved hypothetical protein	5.5	-	
RB3277	221	hypothetical protein	10.4	-	
RB3362	87	hypothetical protein	11.9	-	
RB3366	78	hypothetical protein	5.6	+	
RB3394	36	hypothetical protein	10.5	+	
RB3399	65	hypothetical protein	9.7	-	
RB3575	152	membrane protein	10.1	+	[21]
RB3603	344	secreted protein	4.6	-	
RB3675	742	secreted protein	8.4	+	
RB3688	53	hypothetical protein	9.3	-	
RB3880	82	hypothetical protein	10.7	-	
RB3953	857	hypothetical protein	5.2	-	
RB3981	161	hypothetical protein	4.1	+	
RB3994	191	hypothetical protein	4.1	+	
RB4097	733	conserved hypothetical protein	6.2	+	
RB4145	90	hypothetical protein	12	-	
RB4194	53	hypothetical protein	11.4	-	next to a seronine/threonine kinase
RB4269	282	glutamic acid specific endopeptidase	5.6	-	
RB4358	123	hypothetical protein	6.5	-	[24,66]
RB4373	109	hypothetical protein	4.8	-	
RB4657	123	hypothetical protein	12.2	+	
RB4951	95	hypothetical protein	12.1	+	
RB5262	95	membrane protein	6.3	+	
RB5409	97	hypothetical protein	12.7	+	
RB5415	62	hypothetical protein	12.3	+	
RB5745	130	hypothetical protein	10.7	-	genetic information processing
RB6092	361	Peptidase M50	9.6	+	
RB6158	142	hypothetical protein	6	-	
RB6174	69	hypothetical protein	10.6	-	
RB6276	105	Histone-like bacterial DNA-binding protein	10.4	-	
RB6634	365	protein containing DUF1559	5.3	+	
RB6699	47	hypothetical protein	11.1	+	
RB6766	55	hypothetical protein	12	+	
RB6849	101	hypothetical protein	12.8	-	
RB7042	91	hypothetical protein	10.4	+	
RB7116	59	hypothetical protein	11.7	+	ribosomal machinery
RB7117	181	Ribosomal protein L35	11.4	-	
RB7557	327	von Willebrand factor type A domain protein	4.9	+	
RB7646	62	hypothetical protein	10.5	+	
RB7647	73	hypothetical protein	7.4	+	
RB7837	286	Ribosomal protein L2	11.8	+	
RB7838	89	Ribosomal protein S19/S15	10.8	+	
RB7839	119	Ribosomal protein L22/L17	11	+	
RB7840	236	30S ribosomal protein S3	10.4	+	
RB7841	138	Ribosomal protein L16	11.1	+	
RB7849	108	Ribosomal protein S17	10	+	
RB7850	122	Ribosomal protein L14b/L23e	11	+	
RB7852	196	50S ribosomal protein L5	10.4	+	
RB7854	61	Ribosomal protein S14	11.8	+	
RB7856	181	50S ribosomal protein L6	10	+	
RB7857	149	Ribosomal protein L18P/L5E	11.6	+	
RB7859	177	Ribosomal protein S5	10.6	+	
RB7894	398	translation elongation factor EF-Tu	5.2	+	[24,66]
RB7899	141	50S ribosomal protein L11	9.6	+	
RB8119	142	hypothetical protein	10.1	+	
RB8457	113	hypothetical protein	11.5	-	
RB8594	41	hypothetical protein	9.2	+	
RB8669	37	hypothetical protein	11.5	+	ribosomal machinery
RB9343	59	hypothetical protein	11.4	+	
RB9417	103	hypothetical protein	10.5	+	
RB9460	79	hypothetical protein	10.3	-	
RB9872	67	hypothetical protein	5.4	+	cell division related
RB10581	384	secreted protein containing DUF1559	6.2	+	[24]
RB11287	75	hypothetical protein	9.1	+	
RB11392	148	conserved hypothetical protein	5	+	
RB11490	181	conserved hypothetical protein, membrane	10.3	+	
RB11707	83	conserved hypothetical protein	9.8	+	stress function
RB11766	129	hypothetical protein	10.4	+	[46]
RB12193	36	hypothetical protein	7.5	-	overlapping with asnB (RB12191)
RB12251	567	RNA polymerase specialized sigma factor	9.4	-	
RB12327	686	TGF-beta receptor, type I/II extracellular region	4.5	-	
RB12329	110	conserved hypothetical protein, membrane	4	+	
RB12396	57	hypothetical protein	11.3	-	
RB12454	199	hypothetical protein	10.3	-	
RB12818	163	conserved hypothetical protein	11.7	-	ribosomal machinery
RB12821	117	Ribosomal protein L19	11.1	-	
RB12824	146	Ribosomal protein S16	5.3	-	
RB12837	65	hypothetical protein	9.8	+	ribosomal machinery
RB12839	225	Ribosomal protein L1	9.8	+	

### Experimental design and array data quality assessment

The experimental conditions used were chosen to mimic the natural environment of *R. baltica; *however, stress conditions were constrained by the detection limit of the microarray technology used and, hence, were required to elicit a sufficiently pronounced response from the organism. In contrast to steady-state or single-time-point studies, time series experiments can show the dynamic of gene expression.

The negative, positive and stringency controls printed on the array gave no indications for unspecific hybridizations. Co-hybridizations of two cDNA samples prepared from the same total cellular RNA (self-self hybridization) suggested that genes with an expression log ratio value greater than 1.5 and smaller than -1.0 for heat and cold shock, respectively, could be regarded as differentially expressed. Salt stress log ratio values over 1.2 and below -1.0 were considered as significant.

### Effect of stress on *Rhodopirellula baltica*

No growth was detectable during stress conditions nor were any obvious morphological changes by microscopic investigation. Under optimal conditions *R. baltica *has a doubling time of 10-12 hours [21], suggesting physiological effects are not measurable during the short stress period of, at maximum 5, hours.

### Specific results of the shift experiments

#### Heat shock

In their natural environment *R. baltica *cells can be regularly exposed to higher temperatures, for example, due to irradiation at the water surface. Therefore, *R. baltica *cells were rapidly shifted from 28°C to 37°C and observed over a period of 300 min in the first experiment. This is approximately 9°C above the optimal growth temperature reported by Schlesner *et al*. [2]. Employing a higher temperature is very likely to kill the cells. The time series reveals a quick response of *R. baltica *to sudden temperature up-shifts. In total 2372 genes are regulated out of which 1140 genes encode hypothetical proteins. 390 genes (5%) were regulated after 10 min. This number increased to 750 genes (10%) after 300 min (FIGURE 1i). The COG classes containing the translation [J] and amino acid transport and metabolism [E] were the largest down-regulated classes. Up-regulated genes were assigned to the COG classes of replication, recombination and repair [L], post-translation modification, protein turnover and chaperons [O], transcription [K], secondary metabolites biosynthesis, transport and catabolism [Q], cell envelope biogenesis, outer membrane [M] and general function prediction [R] (FIGURE 2i).

Taking a closer look at the response of *R. baltica *to thermal stress revealed the induction of many known heat shock proteins (Hsp): ClpB (RB6751), GroEL (RB8970), DnaJ (RB8972), GrpE (RB8974), Hsp20 (RB10279, RB10283), dnaK (RB9105), as well as the ATP-dependent protease ClpP (RB9103). Also up-regulated were the chaperonins Cpn10 (RB10627 and RB8969) and Cpn60 (RB8966) as well as the cell division protein FtsH (RB2966) (Cluster 4 in ADDITIONAL FILES 2). Previous proteomic studies found the proteins of these genes as well, except FtsH, DnaJ and Hsp20 [22,24].

The regulation of the heat shock response in *R. baltica *involves many transcriptional regulators. *TetR *(RB838) and *GntR *(RB1862, RB8695) showed an up-regulation, which affirms their important role in early heat shock response [34]. A gene encoding for *GntR *was also found in the environment on the planctomycete fosmid 3FN from a Namibian coast metagenome study [18]. In *E. coli *the induction of the majority of heat shock genes results from a rapid and transient increase in the cellular level of an alternative 32-kDa sigma factor (sigma32) encoded by *rpoH *along with the alternative sigma factors E and 54, encoded by *rpoE *(RB2302) and *rpoN *(RB6491), respectively [27]. Although, all genes are present in the *R. baltica *genome, they were not observed to be regulated, suggesting a significantly different response cascade.

*R*. *baltica *also showed an extracytoplasmic stress response. The gene coding for *SecA *(RB11690), belonging to the *Sec *system, was induced. This indicated an activation of protein translocation, most probably from the riboplasma to the paryphoplasm or medium. Proton channels were induced and motility was inhibited as the flagellar motor switch protein (*Fli*G - RB12502) was down-regulated after 20 min. This was followed by the inhibition of the type 4 fimbrial assembly protein (*pilC *- RB11597) after 40 min.

#### Cold Shock

To investigate the response to cold shock, *R. baltica *cells were shifted from the optimal growth temperature 28°C [35] to 6°C and observed for a period of 300 min. 6°C was chosen for this study as this is a common temperature in the Baltic Sea. Sudden temperature chances occur naturally due to turbulences between water layers. Further, the temperature difference of 22°C is generally regarded as standard for cold shock studies with bacteria [27,36]. Compared to heat shock only one third (922) of the regulated genes were differentially expressed. Out of these 922 regulated proteins, 391 genes (42%) encode for hypothetical proteins. The cold shock response reached its peak after 20 min with 419 differentially expressed genes (6%) and decreased thereafter (FIGURE 1ii). In contrast to the heat shock experiment, it seemed that *R. baltica *needed approximately one hour to adapt to cold conditions. Like other bacteria, *R. baltica *responded to cold conditions with the up-regulation of genes coding for stress response [COG class O], cell envelope and transport [M], transcription factors and solute uptake. Genes for amino acid biosynthesis [E] as well as protein fate and synthesis [J] were down-regulated (FIGURE 2ii) [28].

Transcriptional activity was regulated by the up-regulation of diverse RNA polymerase sigma factors, such as *rpoD *(RB6780) and *sigK *(RB1392). A homolog of *rpoD *(RB6780) was also found on the planctomycete fosmid 13FN [18]. 20 min after the exposure of *R. baltica *to cold stress conditions it started to express genes implicated in the modification of cytoplasmic membrane composition, fluidity as well as morphology. The alteration of the lipid composition in the cold has been previously reported in other microorganisms [37]. In *R. baltica *genes coding for cell envelope (RB6114 and RB6895), transport (RB4870), lipid metabolism (RB316) and 18 genes coding for membrane proteins were repressed after 20 min.

Furthermore, *R. baltica *repressed genes involved in sporulation *oppB *(RB12861) and O-antigen flippase (RB2503), *flaA *(RB4454) and pilus assembly (RB4061 and RB5478), leading to reduced motility and budding ability. Genes associated with amino acid biosynthesis, especially with synthesis and fate of glutamine (RB4269) and glutamate (RB5653) were also affected. The latter have been shown to be translated [22,24]. A glycosyltransferase (RB12831) and glycosidases (RB2988, RB2990 and RB2991) were up-regulated at 300 min probably to aid in cell wall remodeling.

Although, incorrect protein folding at low temperature is less expected than at high temperatures, chaperons and proteases are required to deal with intracellular protein perturbations [28,38]. Here, this was observed in the induction of *GroEL *(RB8970) [22,24] and *htr*A-protease (RB12752). One of the most prominent responses of microorganisms to cold shock is the induction of cold shock proteins. However, the two annotated cold shock proteins of class I (*CspA *- RB4681 and *Cspl *- RB10009) [39,40] were not observed to be regulated. One may hypothesize that the stabilization of RNA in *R. baltica *employs a different protein compliment than observed in *E. coli*.

#### High salinity

As a marine organism, *R. baltica *must adjust to the haline stratification of the Baltic Sea [41,42]. While moving through the water column *R. baltica *cells are exposed to variable concentrations of dissolved salts. In general, an osmotic up-shift forces bacteria to change their physiology by activating or deactivating specific enzymes or transporters, in order to maintain osmotic balance [43]. To gain an understanding of the genetic events that occur during the early stages of salt adaptation, *R. baltica *cells were subjected to salt up-shock from 17.5‰ salinity (Baltic Sea) to 59.5‰ (hyper saline environment). Previous experiments have shown that *R. baltica *is able to grow between salinities of 4.2‰ and 59.5‰ [2] and does not grow at salinities over 90‰ (Wohlrab, unpublished data).

In total, 1127 genes showed differences in gene expression over the whole time series. 656 of these genes (58%) were annotated as hypothetical proteins. The salt up-shock results indicated an increase in the number of regulated genes over time. After 10 min, 61 genes (1%) were regulated. The largest number (543 - 8%) was observed at 300 min (FIGURE 1iii). *R. baltica *cells seem to adapt slowly to high salt concentration. This might be a result of the cell compartmentalization and resulting ability of *R. baltica *to temporarily resist higher salt concentration without notable cellular responses.

The response of *R. baltica *to salt stress includes repression of genes associated with the following COG classes: induction of amino acid transport and metabolism [E], lipid metabolism [I], transcription [K], translation process [J]. Induced genes were involved in classes of the heat shock experiment (discussed above): [O], [M] and [L]. In addition, genes in the energy production [C] and cell division and chromosome partitioning [D] classes were induced (FIGURE 2iii). Similar to other bacteria, *R. baltica *accumulated glutamate and trehalose as cytoplasmic osmoprotectants in response to osmotic stress [44]. Glutamate dehydrogenase (RB6930) showed an up-regulation after 10 min and was also present in the proteome [24]. Trehalose synthetase *treS *(RB519) was induced after 60 min. Cysteine, as a general protective component, was only needed in the first hour in elevated salt concentrations and was repressed afterwards (RB4386).

The accumulation of compatible solutes is a widely distributed mechanism used in coping with changing salinity concentrations [44,45]. In *R. baltica *74 planctomycetes-group-specific genes are annotated as hypothetical proteins carrying a Domain of Unknown Function (DUF1559) [18]. This domain belongs to a new family of solute binding proteins (PF07596) [46] and was also found on the planctomycete fosmid 8FN [18]. Nine of these genes were up-regulated during the first hour of the cold and salt shock experiments. During the heat shock experiment, 16 of these genes were down-regulated. In vitro experiments have shown that some of these compatible solutes also possess general protein stabilization properties in addition to their osmoprotective property [47]. These homologous proteins do not play an integral role in the transport process per se, but probably serve as receptors that trigger or initiate translocation of solutes through membranes by binding external sites of the integral membrane proteins of the efflux system. In addition, some solute-binding proteins function in the initiation of sensory transduction pathways [46].

*R. baltica *up-regulated an efflux pump (RB7603) and a Na^+^/H^+ ^antiporter (RB1433) 300 min after salt shift. Both may play a role in the active export of salt ions out of the cells. Quinone oxidoreductase-like protein (RB10967), induced after 40 min, had been implicated in respiration-coupled Na^+ ^efflux as also shown in *D. vulgaris *[30]. Regulatory proteins like sigma-54 factor *rpoN *(RB6491), *rpoA *(RB12626) and *rfaY *(RB12251) were down-regulated. *rpoN *and *rpoA *were found to be translated [22,24]. *R. baltica *inhibited the genes for cell division (*soj *- RB2291) and chromosome segregation (SMC - RB6065) after 60 min salt stress, as well as diverse transferases (RB12080, RB8898, RB12690, RB2498, RB8222, RB9617) involved in the cell envelope modification. Interestingly, the pilin transport apparatus and the thin-pilus basal body (*pilM *- RB2860 and *pilT *- RB12773) were induced after one hour as were principle pilus associated adhesion (*pilC *- RB12781) and *pilB *(RB12774). Genes coding for biopolymer transport proteins (*exbB *- RB12053 and *exbD *- RB12055) were also induced. A homolog to *exbD *was annotated on the planctomycete fosmid 3FN [18]. It is known from studies of other organisms that genes encoding the flagellar and chemotaxis systems are up-regulated to move away from the stressful cations [30]. However, none of the flagellar genes were regulated and the genome does not harbor any essential chemotaxis genes except *cheY *[16]. Notably, the survival protein (*SurE *- RB10258) and two genes coding for the mechanosensitive ion channel (MscS - RB12279 and RB10255) were induced. The latter provides protection against hypo-osmotic shock, responding both to stretching of the cell membrane and to membrane depolarization [48]. Genes in Cluster 22 (ADDITIONAL FILE 2) seemed to be significantly affected by salt stress only.

### Common stress response

*R. baltica *showed a common stress response to all three tested environmental factors. Several known general stress genes were induced, such as genes coding for the manganese-containing catalase (RB10727), which is also present in the proteome [21,22,24]. Ferritin and *Dps *(RB4433) or pyridoxamine 5'-phosphate oxidase (RB4438) belong to a general stress cluster (RB4432-4438) and were initially described by Hieu *et al. *[24]. Thioredoxin (RB10378) could serve as an electron donor for the up-regulated methionine sulfoxide reductase gene (*msrB *- RB2268) [49,50]. The genes could be regulated via *rpoN *found on the proposed upstream sigma 54-dependent promoter (RB10378) [51].

Perhaps to cope with reactive oxygen species (ROS), typically present under stressful conditions [50], the nitrogen fixation protein (*nifU *- RB3596) was induced. *NifU *is involved in the biosynthesis and repair of ROS scavenging iron-sulfur clusters. Finally, the peptidase M50 (RB6092) may have been induced to regulate stress response, sporulation, cell division, and cell differentiation [52].

Genes involved in *R. baltica's *fatty acid metabolism - for example, oA-acyl carrier protein transacylase (*fabD *- RB314), the acyl carrier protein (*acpP *- RB318) and the *fabB *(RB320) gene - were repressed under all conditions.

Interestingly, the machinery for the rearrangement and interchange of genetic material was induced under all three stressful conditions. It seems to play an important role in the organism's long-term adaptation. *R. baltica *harbors 81 non-randomly distributed transposases in its genome. Notably, under heat stress three times more transposase genes were up-regulated than under cold stress and twice as many as under salt stress. Shared induction shows five IS3/IS911, three ISXo8, two putative transposases (RB170, RB5888, RB11749, RB11802, RB12940, RB2186, RB9907, RB12239, RB934 and RB7389), and one integrase (RB11750). Rearranging the genome to select the most efficient gene combination has been described as a common way to adapt quickly to extreme environments [34]. Relaxed DNA may also be required to get better access to the gene regions for increased expression. Here, DNA relaxation is suggested by the repression of histone-like DNA-binding protein (RB6276).

In line with an alternative global sensing and regulation system initially proposed by Glöckner *et al*. [16], a common pattern concerning sensing and regulation response was detected. *R. baltica *contains 37 genes belonging to the extracytoplasmic function (ECF) subfamily of sigma 70 [53]. The genes RB138, RB13241 and RB10049 are up-regulated under all three stress conditions. Studholme *et al*. [46] suggests that ECF-factor RB10049 is the regulator for the conserved hypothetical protein RB10051. The conserved domain belongs to a new group of proteins that share novel domains referred to as planctomycete-specific (PSD) or planctomycete-specific cytochrome C (PSC). RB10051 contains the PSD1 (DUF1553 - PF07587) and PSC2 (DUF1549 - PF07583) domains, suggesting a function in redox reactions [46]. Each domain is represented 41 times in the whole genome of *R. baltica *[18].

Additionally, at 300 min the ECF-sigma factor RB138 was up-regulated together with serine/threonine protein kinase (RB140). Protein kinases are believed to be involved in stress response [37,54]. The serine/threonine protein kinase (RB12942) and two histidine-kinases (RB4511 and RB10330) were up-regulated during heat shock. Whereas, under cold shock only one serine/threonine kinase (RB8505) was induced. Under salt stress a histidine-kinase (RB13122) and three two-component systems (RB5780, RB12952 and RB13118) were induced.

Finally, the ECF-sigma factor RB1790 was up-regulated, but only under high salinity conditions. In summary, the results confirmed that ECF sigma factors, as well as two-component systems, are heavily involved in stress sensing and regulation of *R. baltica*. The importance of these genes in the natural environment is asserted by the presence of a homolog to RB12952 on the planctomycete fosmid 6N14 [18].

The down-regulation of genes associated with the ribosomal machinery (55%) was observed. During heat shock and high salinity these genes were permanently repressed, whereas under cold shock they were only repressed within the first hour. Of the 51 ribosomal proteins in the whole genome, 18 genes encoding proteins of the small- and large subunit (RB1233, RB12821, RB12824, RB12839, RB7117, RB7837 - RB7841, RB7849, RB7850, RB7852, RB7854, RB7856, RB7857, RB7859 and RB7899) were repressed. Additionally, a set of genes involved in RNA metabolism, protein synthesis, as well as *R. baltica's *only translation elongation factor (EF-Tu - RB7894) were repressed. The genes for the conserved hypothetical protein RB12818 and the hypothetical protein RB12837 were co-regulated which suggests an association with the translation machinery. The repression of the ribosomal genes, along with a large set of genes involved in RNA metabolism, protein synthesis, cell growth (Cluster 1 ADDITIONAL FILE 2), has been reported as a general feature of the environmental stress responses (ESR) [33]. It has been assumed that they are acting as stress sensors [55]. This coincides nicely with the induction of the ribosomal proteins at 300 min under cold shock conditions. Recovery and ongoing adaptation of *R. baltica *was further supported by the up-regulation of the ribosomal-binding factor *rbfA *(RB5503), which is, aside from *csdA*, required for optimal growth at low temperatures [56].

### Hypothetical proteins

Approximately 50% of the regulated genes observed have no known function in each of the three environmental stress experiments. Some of these share a similar expression profile (ADDITIONAL FILE 2). We propose that some of these genes are involved in cell morphology changes, stress sensing and regulation. The low number of known transcriptional regulators (2.4%) in the genome of *R. baltica *[53], coupled with the fact that most of the essential pathways encoded are not organized in operon structures [16] support the hypothesis of novel global regulation mechanisms. Hypothetical proteins that carry regulatory domains, like the FHA domain in RB1789 or a putative transcriptional regulatory domain in RB9999 are strong candidates. RB11766 might regulate the gene next to it, which is a so called giant gene (RB11769) [57]. This giant gene encodes a novel peptide motif that is most likely involved in cell morphology changes [46]. The importance of the hypothetical proteins RB11505, RB10954, RB10956 and RB10958 was further supported by their presence on the proteome gels of Hieu *et al. *as well as Gade *et al. *[21,22,24]. The latter three of these genes were claimed to be among the most abundant proteins in *R. baltica *cultures grown on mineral medium.

### *Planctomycete *special feature: Genes encoding sulfatases

The genome of *R. baltica *contains no less than 110 sulfatases. It is assumed that they are involved in the recycling of carbon from complex sulfated heteropolysaccharides. Although the mineral medium does not contain any sulfated polysaccharides, we found 11 sulphatase genes were up- or down-regulated (TABLE 3) during the different stress experiments. These included one choline sulphatase (RB1205), seven arylsulfatases (RB13148, RB1477, RB3403, RB406, RB5146, RB684 and RB9498), two sulphatase genes without specificity (RB3956, RB5294), and one alkylsulfatase (RB11502). Furthermore, during life cycle experiments (unpublished data) we found evidence that certain sulfatases are only regulated in specific growth stages, which could indicate their involvement in the remodeling of the distinct morphological features of *R. baltica*. Sulfatase genes RB1477, RB5294, RB9498 and RB11502 were induced. We propose that RB9498 and RB11502 have an extracellular function and may be involved in the formation of an extrapolymeric substance.

**Table 3:** Differentially expressed sulfatase genes of *R. baltica *are shown

**ID**	**Product**	**AA**	**Signal P**	**Heat**	**Cold**	**Salt**	**Remarks**
RB1205	choline sulfatase	456	0.80	repressed	repressed		
RB3403	arylsulfatase precursor	491	0.99	repressed	repressed		[22,24]
RB3956	sulfatase	489	0.98			repressed	
RB5146	arylsulfatase A precursor (ASA)	522	0.95	repressed			
RB9498	arylsulfatase A	518	0.97	induced			[22,24],
RB11502	alkyl sulfatase or beta-lactamase	445	1.00			induced	
RB1477	arylsulfatase precursor	538	0	induced		induced	
RB5294	sulfatase	533	0			induced	wall* unpublished results
RB406	arylsulfatase	557	0	repressed	repressed		
RB684	arylsulfatase precursor	653	0	repressed		repressed	life cycle unpublished results
RB13148	arylsulfatase A [precursor]	1012	/	repressed			life cycle unpublished results

Six sulfatase genes (RB406, RB684, RB1205, RB3403, RB5146 and RB13145) were repressed after 300 min of heat shock. They may have been involved in the rearrangement of the cell wall formation, which comprises a protein sacculus with disulfide bonds [12]. In summary, these results show the diverse roles that sulfatases may have and, furthermore, that only a variety of different experimental approaches will increase our knowledge of these roles.

## Conclusion

This work presents the first transcriptome study of the environmental stress response of a marine, free-living *Planctomycete*. Although *R. baltica *is an unusual organism in many aspects, its stress responses to heat and cold shock as well to changing salinity were in line with earlier results reported for other model organisms. Heat shock induced a set of chaperons, likely to protect cellular proteins from denaturation and breakdown. Growth in the cold may be followed by the induction of genes altering lipid metabolism. Salinity shifts resulted in the activation of planctomycete-specific groups of genes including genes involved in morphological change and an extracytoplasmic stress response. All stressors triggered the down-regulation of the ribosomal machinery, the up-regulation of transposases and the induction of several ECF-sigma factors and two-component systems. This supports the hypothesis that *R. baltica *is regulating its gene activity on a global rather than operon level. Aside from well characterized stress response genes, about 2000 genes of unknown function, constituting 30% of the genes predicted in the genome, were affected. This, combined with proteome studies and the presence of some of the genes in fosmid libraries, provides a strong indication that the vast number of genes with unknown function play a vital role in the organism's environmental response. The regulation of 11 sulfatases during stressful conditions suggests that these genes are heavily involved in the core cellular function of *R. baltica*. The data presented lead to the conclusion that *R. baltica's *rich repertoire of genes is combined with a fine tuned regulation mechanism to best respond to the changing conditions of its habitat. Nevertheless, data analysis has just started and further investigations concerning the genes involved in the life-cycle, the stress response pathways, promoter regions and network analysis are already ongoing or planned for the near future.

## Methods

### Bacterial growth conditions

For all experiments *Rhodopirellula baltica *SH1^T ^cells were grown as chemostat cultures in a mineral medium containing 10 mM glucose as the sole carbon source and 1 mM ammonium chloride as a nitrogen source at 28°C [20]. Chemostat (Ø 13.5 cm × 25 cm, 1 l, Schott, modified by Ochs, Bovenden) parameters used were: pH 7.4, average dilution rate 0.75 ml/min and pO_2 _around 100%. The cultures had an OD_600 nm _of 0.5 - 0.6 (corresponding to log phase). The cells were harvested after 5 dwell times.

### Sample collection, cell lyses, RNA Isolation and cDNA synthesis

After harvesting the *R. baltica *cultures, an aliquot was collected to serve as the time-zero reference. The culture broth was collected in 500 ml tubes and swirled briefly in an ethanol-dry ice bath to rapidly cool the cultures and prevent shifts in the RNA profile. Subsequently, the broth was centrifuged at 6000 rpm for 20 min at 4°C (Beckman Coulter™ AvantiTM626 J-20XP, JA10 Rotor). The pellets were re-suspended in 0.1 M Tris-HCL and then re-centrifuged. Cell pellets were shock-frozen in liquid nitrogen and stored at -80°C. Total RNA was isolated using the protocol of the TRI Reagent^® ^Kit by Ambion (Austin, USA). The purity and quality of the extracted total RNA was checked with an Agilent 2100 Bioanalyzer (Agilent Technologies, Palto Alto, USA) and gel electrophoresis. cDNA synthesis was performed using the SuperScript direct cDNA labeling kit by Invitrogen (Karlsruhe, Germany) according to the manufacturer's instructions with random hexamers and unlabeled dCTP/dUTP, followed by a three hour reverse transcription incubation step at 46°C. The RT reaction was halted by incubation for 3 min at 95°C. To hydrolyze the RNA, 0.1 M NaOH was added, incubated at 65°C for 15 min and neutralized with 0.1 M HCL. The remaining cDNA was precipitated overnight at -20°C and the pellet washed with 70% Ethanol.

cDNA was directly labeled using the Platinum *Bright*™ nucleic acid labeling kit based on KREATECH's patented Universal Linkage System (ULS) (Biocat, Heidelberg, Germany) according to the manufacture's protocol.

Concentrations of RNA and cDNA were measured, and incorporation of the dyes Alexa 546 and Alexa 647 were checked using a Nanodrop ND-1000 spectrophotometer (NanoDrop Technologies, Wilmington, USA).

### Experimental design and sample preparation

In three independent hybridizations conducted for each experiment and time point, the expression profiles of cells that had undergone stress were compared with those of cells at time zero. That is, the array analysis of each Alexa 647 labeled sample was compared with those of Alexa 546 labeled time-zero samples. The data shown are based on the analysis of all three replicates performed for each of the conditions.

Samples for expression profiling and microscopic analysis were collected at 10, 20, 40, 60 and 300 min in all three stress experiments.

### Heat shock from 28°C to 37°C

Cells grown continuously at 28°C were collected by centrifugation. An aliquot was removed for RNA extraction and taken as the time zero reference for the heat, cold and salt stress experiments. Aliquots were re-suspended in an equal volume of 37°C medium and returned to 37°C for cultivation.

### Cold shock from 28°C to 6°C

Cells grown continuously at 28°C were collected by centrifugation, re-suspended in an equal volume of 6°C medium and returned to 6°C for cultivation.

### Salt stress from 17.5‰ to 59.5‰ salinity

Similar to the heat and cold shock experiments, an *R. baltica *culture was grown in mineral media with 17.5‰ salinity. Cells were harvested and aliquots were transferred to a mineral media with a salinity of 59.5‰.

### Whole Genome Array construction, hybridization and image analysis

The whole-genome oligonucleotides for *R. baltica *SH1^T ^(*Pirellula *AROS 630 Version 1.0) were purchased from Operon (Cologne, Germany) and diluted to 20 μM concentration in Micro Spotting Solution Plus spotting buffer (Telechem, Sunnyvale, USA). Spotting was done with three replicates per gene, per slide onto GAPS II aminosilane slides (Corning, Schiphol-Rijk, Netherlands) using a SpotArray 24 spotting device (Perkin Elmer, Wellesley, USA) together with 48 Telechem Stealth Pins (Telechem, Sunnyvale, USA). The arrays were subsequently exposed at 245 nm and 360 mJ in the GS Gene Linker (Bio-Rad, München, Germany), followed by incubation at 80°C for at least 3 h. Slides were stored at room temperature in the dark until use.

Blocking, denaturing, hybridization, washing and N_2 _drying procedures were carried out in an automated hybridization station HS400 (Tecan, Crailsheim, Germany). The spotted arrays were blocked in prehybridization solution containing 250 mM NaCl, 5 mM Tris/HCl at pH 8.0, 50% formamide, 0.5 × SSC, 0.05% BSA, and 1% blocking reagent from Roche Diagnostics, Mannheim, Germany for 45 min at 52°C. For hybridization at least 2 μg of Alexa 546 dye-labeled and 2 μg of Alexa 647 dye-labeled total cDNA were combined and taken up in a final volume of 100 μl DIG Easy Hyb hybridization solution (Roche Diagnostics, Mannheim, Germany). After the blocking step, the sample solution was applied to the arrays, denatured at 95°C for 3 min and hybridized under stringent conditions at 52°C for over 12 hours. After hybridization slides were washed at room temperature in ULTRArray Low Stringency Wash Buffer (Ambion, Austin, USA) and dried by N_2_.

### Signal detection and data analysis

Slides were scanned at a resolution of 5 μm using a ScanArray Express Microarray scanner (Perkin Elmer, Wellesley, USA) with varied laser power and photomultiplier tube (PMT sensitivity) for each slide. The accompanying image analysis software, ScanArray Express Version 4.0, was used for automatic spot detection and signal quantification of both fluorophores. Raw data were automatically processed using the microarray data analysis software tool MADA , developed in-house. Firstly, the spot intensities were corrected for local background (mean spot intensity minus mean spot background intensity). Signals were only assessed as positive if mean spot pixel intensity was higher than the mean local background intensity plus twice the standard deviation of the mean local background pixel intensity. Each gene is spotted in three replicates. Spot replicates with poor quality were removed from the data set according to MADA's outlier test results. This test first computes the standard deviation of all replicates. Secondly, one replicate is omitted and the standard deviation is recalculated; if the deviation differs more than 50% from the previous deviation, the omitted replicate is regarded as an outlier. This procedure is repeated for all replicates

Expression is described through the ratio and intensity, where R is the fluorescence log ratio of the experiment time point relative to the control condition (e.g. R = log2 (result of channel 10 min/result of channel control/reference)) and I is the log mean fluorescence intensity (e.g. I = log10 (result of channel 10 min × result of channel control/reference)).

Each data point represents a regulation factor (ratio) in a logarithmic scale for one gene calculated from the positive replicates for a particular probe coming from two RNA pools (reference and sample). Normalization was carried out by LOWESS fitting on an R-versus-I plot with a smoothing factor of 0.5. Each time point of the time-series experiment was hybridized independently three times. The expression data (ratio) of the three hybridizations were combined to one expression data point (ratio) by averaging and the standard deviation of the average value was calculated. Only ratios with a standard deviation less than 25% were regarded as genes that are regulated. Differentially expressed genes were detected by a fixed threshold cut off method (i.e. a two-fold increase or decrease) based on the results of self-self hybridization. Using the same biological sample, the reference (untreated sample) is labeled twice, once with Alexa 546 and once with Alexa 647, and the variability between the two sets of measurements is calculated to estimate the experimental noise. Ideally, there should not be any variability and all expression points should have a ratio close to zero. In reality, however, this is never the case and thresholds based on the distribution of these data along the y-axis were defined for the further experiments.

Consequently, *R. baltica *genes detected with intensities resulting in ratios above or below these thresholds can beregarded as up- or down-regulated.

### Cluster analysis

Differentially expressed genes present in the complete time course profile (10, 20, 40, 60 und 300 min) for all three experiments were clustered using the k-means clustering approach (Euclidean distance metric, k = 30 clusters and 49 (max. 500) iterations) [58] with the software tool Multiexperiment Viewer MeV Version 4.0.2 from the TM4 microarray software suite [59]. Briefly, the clustering algorithm arranges genes into a given number of clusters, k, according to similarity in their expression profiles across the entire array experiments, such that genes with similar expression patterns are clustered together. The data are displayed in tabular format where each row of colored boxes stores the variation in transcript abundance for each given gene and each column stores the variation in transcript levels of every gene in a given mRNA sample, as detected on one array. The variations in transcript abundance for each gene are depicted by means of a color scale, in which shades of red represents increases and shades of green represent decrease in mRNA levels, relative to the unstressed culture, and the saturation of the color corresponds to the magnitude of the differences. Black coloration indicates no change in transcript level while grey represents missing data.

### Genome tools

The genome of *Rhodopirellula baltica *was automatically re-annotated based on updated homology searches (June 2005 - MicHanThi [60]). The updated annotation including all tool results is publicly available at [61]. JCoast [62] was used as a tool for the visualization, interpretation, COG-assignment statistics and comparison of genomic data stored in GenDB V2.2 [63]. The Venn diagrams were generated by BioVenn [64].

### Microarray Datasets

Each microarray used in this study contained 7325 known or predicted *R. baltica *genes according to Glöckner *et al*. [16]. A detailed description of the array can be found at the NCBI's Gene Expression Omnibus (GEO) database under accession number GPL7654. The complete microarray datasets covering the expression of *R. baltica *cultures exposed to heat, cold and high salinity, are public available in the GEO repository  under accession numbers GSE13769, G SE13856 and GSE14075 [65].

## List of abbreviations

COG: Cluster of Orthologous Group of Genes; DUF: Domain of Unknown Function; ECF: Extra Cytoplasmic Function; ESR: Environmental Stress Response; FHA: Forkhead-associated; GEO: Gene Expression Omnibus; R.: *Rhodopirellula*; RB: *Rhodopirellula baltica*; ROS: Reactive Oxygen Species; ORF: Open Reading Frame.

## Competing interests

The authors declare that they have no competing interests.

## Authors' contributions

**PW **conceived the study, initiated, conducted the experimental analysis, validated microarray and optimized experimental steps, wrote the manuscript, did the statistical analysis and analyzed the data.

**CK **was involved in the chemostat cultivation of *R. baltica *and statistical analysis and in the biological interpretation of the data

**AE **wrote MADA the microarray analysis tool and designed the microarrays.

**CQ **was responsible for the automatic reannotation of the genome and set up the web access

**PL **and **JH **established the chemostat cultivation of *R. baltica*. **JH **supervised the chemostat cultivation.

**FOG **contributed background information and was involved in writing and finishing the manuscript.

All authors read and approved the final manuscript.

## Supplementary Material

Additional file 1 **List of differentially expressed genes for all three stress experiments**. The data provided represents the differentially expressed genes of *Rhodopirellula baltica *exposed to three stress conditions (heat shock, cold shock and high salinity). Click here for file

Additional file 2 **List of selected clusters used in k-means clustering**. The data provided represents detailed information about the gens and clusters used in the k-means clustering of the stress experiments. Click here for file
